# Actin Dynamics Associated with Focal Adhesions

**DOI:** 10.1155/2012/941292

**Published:** 2012-03-08

**Authors:** Corina Ciobanasu, Bruno Faivre, Christophe Le Clainche

**Affiliations:** Laboratoire d'Enzymologie et Biochimie Structurales, Centre de Recherche CNRS de Gif-sur-Yvette, avenue de la Terrasse, 91198 Gif-sur-Yvette, France

## Abstract

Cell-matrix adhesion plays a major role during cell migration. Proteins from adhesion structures connect the extracellular matrix to the actin cytoskeleton, allowing the growing actin network to push the plasma membrane and the contractile cables (stress fibers) to pull the cell body. Force transmission to the extracellular matrix depends on several parameters including the regulation of actin dynamics in adhesion structures, the contractility of stress fibers, and the mechanosensitive response of adhesion structures. Here we highlight recent findings on the molecular mechanisms by which actin assembly is regulated in adhesion structures and the molecular basis of the mechanosensitivity of focal adhesions.

## 1. Introduction

**Figure 1:**
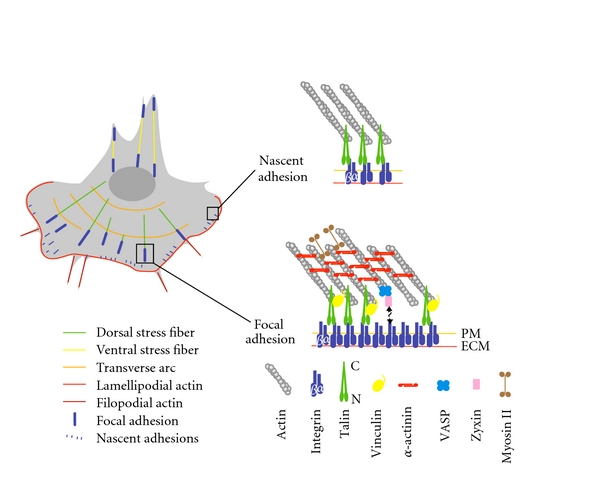
Actin networks in cell migration and organization of nascent adhesions and focal adhesions. Left, scheme of a migrating cell displaying characteristic actin structures: lamellipodial and filopodial actin networks and the three classes of stress fibers (transverse arcs, dorsal stress fibers, ventral stress fibers). Right, actin-binding proteins in focal complexes and focal adhesions. PM, plasma membrane; ECM, extracellular matrix.

In multicellular organisms, cell migration plays an essential role in a variety of physiological processes such as embryogenesis, tissue regeneration, immune response, and wound healing. The misregulation of cell migration is also responsible for many pathological processes including cancers [1]. The migratory cycle is a complex process in which actin dynamics play a central role at every step. Actin assembly drives the extension of flat membrane protrusions called lamellipodia and finger-like protrusions called filopodia to push the membrane. To anchor the protrusion, the cell front interacts with the extracellular matrix (ECM) by forming nascent adhesions (or focal complexes) that are connected to the intracellular lamellipodial actin network. Nascent adhesions can either disassemble or, in response to the actomyosin force, mature into larger structures called focal adhesions (FAs) that assemble contractile actomyosin cables (stress fibers) (Figure 1). To complete this migratory cycle, the contraction of stress fibers retracts the trailing edge [2]. In this cycle, proteins from adhesion structures connect the ECM to the actin cytoskeleton (Figure 1), allowing the growing actin network to push the plasma membrane forward and the contractile stress fibers to pull the cell body and retract the tail. Force transmission to the ECM depends on a variety of parameters including the regulation of actin dynamics in adhesion structures, the contractility of stress fibers, and the mechanosensitive response of adhesion structures [3].

In this paper, we discuss recent findings on the following issues. (1) The mechanism by which actin assembly is initiated in nascent adhesions, (2) the mechanosensitive maturation of FAs, (3) the molecular mechanisms underlying the formation of stress fibers, and (4) the regulation of actin assembly in focal adhesions.

## 2. Initiation of Actin Assembly in Nascent Adhesions

During the migratory cycle, the lamellipodium first interacts with the ECM by forming small and highly dynamic complexes called nascent adhesions (<0.25 *μ*m).

**Figure 2:**
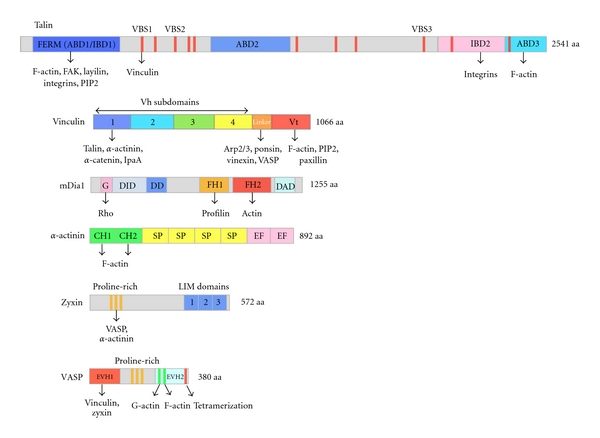
Domain organization of proteins involved in the regulation of actin assembly in focal adhesions. ABD, actin-binding domain; FERM, four-point-one, ezrin, radixin, moesin; IBD, integrin binding domain; VBS, vinculin-binding site (11 VBSs are indicated as red vertical bars); GBD, G-binding domain; DID, diaphanous inhibitory domain; DD, dimerization domain; FH1, formin homology domain 1; FH2, formin homology domain 2; DAD, diaphanous autoregulatory domain; CH, calponin homology; SP, spectrin repeat; EF, EF hand motif; EVH1, Ena/VASP homology domain 1; EVH2, Ena/VASP homology domain 2.

At the molecular level, talin is one of the first proteins involved in the connection between the ECM and the actin cytoskeleton. Talin is a large protein made of a globular FERM domain followed by an elongated rod-shaped domain. The rod domain contains multiple vinculin-binding sites (VBSs). The C-terminal end mediates the formation of a parallel dimer. Talin contains three actin-filament- (F-actin-) binding domains located in the FERM domain (ABD1), the rod domain (ABD2), and the C-terminal domain (ABD3), respectively. Talin contains two integrin-binding domains located in the FERM domain (IBD1) and the rod (IBD2), respectively [4] (Figure 2). Both the early recruitment of talin in nascent adhesions [5] and its requirement for the 2 pN integrin-actin bond in nascent adhesions support the early role of talin [6]. In addition to providing the first ECM-actin link, talin also contributes to adhesion by activating integrin [7].

More recent observations suggest a role for *α*-actinin and myosin-II in the formation of a first template of actin. In this study, myosin-II, like *α*-actinin, acts as a simple crosslinker since the inhibition of its motor activity does not alter this function [8]. In this respect, it is interesting to note that an interaction between *α*-actinin and integrin *β*1 has been described and may contribute to this connection [9]. The physiological relevance of this interaction is unclear since the recent super-resolution imaging of mature FAs indicates that *α*-actinin and integrin *β*1 are too distant to interact [10]. However, such an interaction may exist transiently in nascent adhesions.

Many actin-based processes, including the formation of the lamellipodial actin network, are initiated by a specific signaling-responsive machinery that nucleates new actin filaments in a site-directed fashion. However, none of the actin-binding proteins present in nascent adhesions display a nucleation activity. Therefore, it is very likely that the formation of the first actin meshwork that appears in nascent adhesions results from the capture of preexisting lamellipodial actin filaments.

Alternatively, a recent study showed that, in cells forming filopodia, nascent adhesions form occasionally along the filopodial actin network. Filopodia are very dynamic structures that elongate rapidly before they retract inside the lamellipodial actin network. After retraction, the filopodial actin bundle is used as a precursor for the first actin meshwork of nascent adhesions and the future stress fiber associated with an FA [11].

## 3. The Mechanosensitive Maturation of FocalAdhesions

Nascent adhesions can either disassemble or switch to maturation. In this process the nascent adhesion grows by recruiting a new set of proteins to become a focal complex (<1 *μ*m) and then a focal adhesion (1–5 *μ*m) [12] (Figure 1). Maturation is a mechanosensitive process induced by the actomyosin force generated in the lamella in response to RhoA signalling [13]. In many cell types, the lamella contains contractile stress fibers (see Section 4.2). However, the lamella also exists as a contractile network without remarkable structures [14]. Growing evidence indicates that actin-binding proteins could behave as mechanosensors. However, the mechanisms by which proteins respond to the actomyosin contractility to finally induce the maturation of FAs is still an open issue [15].

To understand the molecular basis of this process, Zaidel Bar and colleagues have systematically compared the composition of nascent adhesions and FAs. The results showed that after talin is recruited in nascent adhesions, the maturation into FAs is accompanied by the accumulation of several proteins including vinculin, VASP, zyxin, and *α*-actinin [5] (Figure 1). Although the formin mDia1 is required for the growth of FAs in response to an external force [16], there is currently no evidence that mDia1 is present in FAs (see Section 5.3).

The description of the spatial organization of proteins in FAs is an important step to understand the sequence of unfolding events that accompanies the maturation process. Recently, the use of three-dimensional super-resolution fluorescence microscopy revealed the vertical organization of these proteins in mature FAs [10] (Figure 1). The N-terminal domain of talin is localized next to the plasma membrane whereas the C-terminal domain extends toward the actin network. Vinculin localizes between the N- and C-termini of talin, in agreement with the presence of numerous (VBSs) along the rod domain of talin. Zyxin localizes 60 nm away from the plasma membrane suggesting the existence of at least one missing intermediate between this protein and the plasma membrane or integrins. The colocalization of zyxin and VASP is in agreement with the zyxin-dependent recruitment of VASP in FAs [10, 17].

A recent proteomic analysis, describing the consequences of myosin inhibition on focal adhesion composition, revealed the extraordinary changes associated with the maturation process. Half of the 905 focal adhesion proteins identified were either enriched or depleted following myosin II inhibition. By analyzing the function in which each protein is involved, this study reveals that myosin II contractility induces a major functional switch by promoting the accumulation of maturation factors and the dissociation of lamellipodial protrusion factors [18].

However, all these proteins are not mechanosensors. In such a process, it is thought that only a few proteins are subjected to a mechanical unfolding to initiate a large chain reaction of protein-protein interactions. The identification of mechanosensitive proteins should be facilitated by the recent development of a calibrated biosensor that measures forces across specific proteins in living cells. The insertion of such a mechanosensor in vinculin showed that high tension across vinculin is associated with adhesion maturation whereas less force is applied on vinculin in disassembling FAs [19].

To identify the mechanosensitive proteins that are subjected to domain stretching or disruption of autoinhibitory interactions, Johnson and colleagues combined the labelling of accessible cysteines with fluorophores in living cells with quantitative mass spectrometry. They showed that one domain of spectrin is stretched upon mechanical stress in red blood cells. Similarly, in mesenchymal cells, the oligomerization state of vimentin and myosin IIa changed upon myosin inhibition [20].

To go deeper into the mechanism by which proteins are mechanically unfolded, single molecule-stretching experiments have been carried out. Del Rio and colleagues used magnetic tweezers to show that the stretching of a fragment of talin induces the binding of vinculin head [21]. However, the relevance of this mechanism for FAs has yet to be determined since, in cells, the myosin-II-dependent recruitment of vinculin also depends on the phosphorylation of paxillin by FAK/Src [22].

## 4. Stress Fibers

### 4.1. Major Components

The actin-binding protein *α*-actinin, that bundles actin filaments, plays a major role in the organization of the actin network in multiple cell-types. *α*-actinins belong to the spectrin group of cytoskeletal proteins. Actin binding is mediated by two calponin homology (CH) domains (Figure 2). *α*-actinin forms an antiparallel rod-shaped dimer with one actin-binding domain at each end. The antiparallel dimerization is mediated by the spectrin repeats of the rod. Cryoelectron microscopy observations suggest that the two CH domains exist in two different conformations allowing *α*-actinin to crosslink actin filaments in a parallel and/or antiparallel fashion [23]. In skeletal, cardiac, and smooth muscles, *α*-actinin isoforms localize in the Z-disc, where they anchor the myofibrillar actin filaments. The contractile bundles of nonmuscle cells such as stress fibers are characterized by alternating bands of *α*-actinin and myosin II [24]. In stress fibers, *α*-actinin separates each actin filament by 15–40 nm. This space allows the insertion of myosin filaments [23, 25]. 

The contractility of stress fibers is mediated by the motor activity of myosin II. The myosin superfamily of actin-based molecular motors consists of at least 25 different classes. The myosin II subfamily includes skeletal, cardiac, and smooth muscle myosins, as well as nonmuscle myosin II. Myosins use the energy of ATP hydrolysis to generate force. Each cycle of ATP binding and hydrolysis is associated with a conformational change of the myosin head—the power stroke—that generates movement [26]. Many myosins are processive motors that never dissociate from the actin filaments. The ability of myosin to walk along actin filaments comes from a tight coordination of the catalytic cycle of each head domain. Myosin II dimer is not a processive motor. The two heads of myosin are not coordinated and the dimer dissociates between each cycle. To generate force, myosin II self-assembles into short bipolar filaments consisting of 10–30 myosin molecules. This self-assembly results in an antiparallel filament of myosin molecules. Hence, the motor domains located at each end of the filament associate with oppositely oriented actin filaments. By pulling actin filaments together, myosin II generates tension [27, 28] ([26] for a review). 

In addition to *α*-actinin and myosin, the F-actin-binding proteins from the tropomyosin family play an important role in the formation of stress fibers [29]. Tropomyosins control the fate of actin filaments in the lamella by selecting the association of appropriate actin-filament-binding partners. Hence, tropomyosin binding to actin filaments prevents Arp2/3-mediated branching and depolymerization by ADF/cofilin [30–32]. A recent examination of the role of six tropomyosin isoforms expressed in U2OS cells indicated that some of them control the stability of actin filaments in stress fibers. Interestingly, Tm4 promotes the formation of stress fibers by inducing the binding of myosin II to mDia2-nucleated actin filaments [33]. 

### 4.2. Formation and Maintenance

Stress fibers of mammalian cells can be divided into three classes based on their orientation in cells and interaction with FAs: ventral stress fibers, dorsal stress fibers and transverse arcs [14]. Each type of stress fiber is assembled by a different mechanism.

Dorsal stress fibers are connected to FAs by only one end (Figure 1). Dorsal stress fibers are actin filament bundles that processively elongate from FAs toward the cell center. Although the RNAi depletion of the formin mDia1/DRF1 led to a decrease in the elongation rate of dorsal stress fibers, this effect could be indirect [34] (see Section 5.3). Live cell imaging in U2OS cells showed that dorsal stress fibers first elongate from FAs to form short filaments containing *α*-actinin [34]. After these *α*-actinin cross-linked bundles reach a length of several micrometers, they connect to transverse arcs (or convert to ventral stress fibers) and they incorporate myosin clusters. By displacing *α*-actinin, myosin generates a periodic pattern. However, since dorsal stress fibers are made of parallel actin filaments, they are likely not contractile. 

Transverse arcs are curved actomyosin bundles parallel to the leading edge of the lamellipodium (Figure 1). Although transverse arcs are not directly connected to FAs, they transmit their force to the ECM indirectly via dorsal stress fibers [35]. These structures flow from the leading edge toward the cell center as a result of myosin activity [14, 36, 37]. In contrast to dorsal stress fibers, transverse arcs form by endwise assembly of short *α*-actinin and myosin containing bundles [34]. A recent study suggested that arcs result from the association of two distinct populations of actin filaments: (1) actin filaments nucleated by the Arp2/3 complex and cross-linked by *α*-actinin and (2) actin filaments nucleated by the formin mDia2/DRF3 that subsequently recruit tropomyosin and myosin II [33]. 

Ventral stress fibers are anchored to FAs at each end [36] (Figure 1). Ventral stress fibers form by the end-to-end association of two dorsal stress fibers or a dorsal fiber with a preexisting transverse arc to form a structure that is anchored at both ends to a focal adhesion [34, 36]. Actin filaments that compose ventral stress fibers exhibit a graded polarity, which allows contractility. 

Live cell imaging revealed that stress fibers have the ability to repair force-induced damages that affect their contractile properties. This process clearly depends on the LIM protein zyxin that is relocated from FAs to stress fibers upon the application of a mechanical stress [38]. This process is also accompanied by the zyxin-dependent relocation of the actin-binding proteins VASP and *α*-actinin. Together with zyxin, these two proteins form a repair complex that accumulates at sites of SF strain and contributes to restore the structural integrity and the contractility of the stress fiber [39]. However, the mechanisms by which the repair complex recognizes strain sites and the mechanism by which VASP and *α*-actinin cooperate to repair stress fibers are not known. In particular, the relative importance of the numerous activities of VASP in this process has not been investigated (see Section 5.1). 

## 5. Regulation of Actin Assembly in Focal Adhesions

As mentioned above, force transmission to the ECM depends on a variety of parameters including the regulation of actin dynamics. FAs contain many actin-binding proteins [3]. The analysis of fluorescent speckles in cells revealed the contribution of individual actin-binding proteins to the force transmission. In particular, vinculin and talin form transient bonds with the retrograde flow of actin filaments, suggesting that they partially transmit force to the ECM [40]. Therefore, the actin-ECM mechanical coupling depends, at least partially, on the association and dissociation rates that characterize the interactions between actin binding proteins present in FAs and the actin network.

The processive elongation of stress fibers from FAs also suggested that force transmission is controlled by actin dynamics [34]. For example, a processive elongator would release the actomyosin tension while a barbed end capping protein is expected to transmit the force efficiently. We showed recently that vinculin inhibits barbed-end elongation [41]. Others found that Ena/VASP proteins promote the processive elongation of actin filaments [42, 43]. However, the mechanisms by which isolated FA proteins and their complexes regulate actin dynamics remain largely unknown.

The following paragraphs describe the mechanisms by which three of these proteins regulate actin dynamics.

### 5.1. Regulation of Actin Assembly by Ena/VASP Proteins

Ena/VASP localize in FAs [44], filopodia, and the leading edge of protruding lamellipodia [45]. Ena/VASP is a family of conserved proteins expressed in vertebrates, invertebrates, and *Dictyostelium discoideum*. These proteins are characterized by the following domain organization: an N-terminal EVH1 domain (Ena/VASP Homology 1 domain) followed by a central proline-rich domain (PRD) and a C-terminal EVH2 domain (Ena/VASP Homology 2 domain). The direct interaction of the EVH1 domain with the FPPPP motifs of zyxin directs the localization of Ena/VASP proteins to FAs [17]. The proline-rich domain mediates the recruitment of profilin. The EVH2 domain contains both a WH2-like domain that interacts with monomeric actin and an F-actin-binding domain (FAB) [46, 47]. Finally the C-terminal end contains a tetramerization motif (Figure 2).

Ena/VASP were first studied for their role in the formation of the actin comet tail that propels the bacteria *Listeria* in the host cell. In this process, VASP interacts with the *Listeria* surface protein ActA which stimulates the Arp2/3 complex to feed the actin tail with branched filaments [3, 48]. Similarly, the binding of VASP to WASP stimulates the formation of Arp2/3-dependent actin-rich protrusions in rat basophilic leukemia cells [49]. The combination of kinetic and biomimetic assays suggested that VASP acts on the catalytic cycle of Arp2/3 activation by favoring the dissociation of the branched filament from the activator of Arp2/3 [50]. The observation that VASP-deficient cells migrate faster with more persistent and slower lamellipodia containing longer filaments suggested a different hypothesis. Bear and colleagues proposed that VASP promotes actin dynamics by protecting actin filament barbed ends against the action of capping proteins [51].

**Figure 3:**
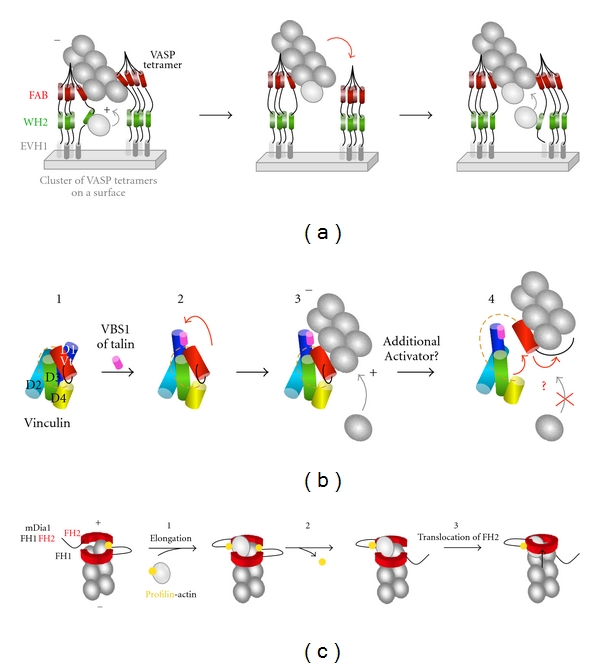
Regulation of actin assembly by VASP, vinculin, and formins. (a) VASP-mediated processive elongation of actin filaments. Clusters of VASP tetramers allow the WH2 domains of several VASP molecules to deliver actin monomers to actin filament barbed ends in a processive manner. This activity is accompanied by an acceleration of barbed end elongation. Between delivery events, VASP remains associated to the side of the filament via its FAB domain, adapted from [42, 43]. (+) and (−) indicate the barbed end and the pointed end of the actin filament. (b) Regulation of actin filament side binding and barbed-end capping by vinculin. In this scheme, subdomains of vinculin are represented as cylinders. The proline-rich region that links Vh and Vt is represented as an orange dotted line. (1) Vinculin is autoinhibited by an intramolecular interaction between Vh (D1 to D4) and Vt. The F-actin binding site located in Vt is masked by D1. (2) The binding of the VBS1 domain of talin disrupts the D1-Vt interaction (red arrow). Vt is unmasked and binds to the side of an actin filament. Barbed end elongation is still possible. (4) The disruption of additional unidentified contacts unmasks the C-terminal arm (black line) of Vt which caps the barbed end of the filament, adapted from [41]. (c) Formin-mediated processive elongation of actin filaments. In this scheme, only the FH1 (black line) and FH2 (red) domains are represented. (1) Addition of a profilin-actin subunit to the formin-bound barbed end. Each FH2 protomer of the formin dimer makes two contacts with the terminal actin subunits at a barbed end. (2) Profilin dissociates. (3) The translocation of one FH2 protomer releases one of the two actin-formin bonds to allow the addition of next profilin-actin subunit, adapted from [3].

More recently, in vitro studies proposed a mechanism in which the clustering of VASP tetramers allows the WH2 domains of several VASP molecules to deliver actin monomers to actin filament barbed ends in a processive manner [42, 43]. This activity is accompanied by an acceleration of barbed end elongation. Between delivery events, VASP remains associated to the side of the filament through its FAB domain. VASP-bound barbed ends are protected from capping proteins (Figure 3(a)). TIRF microscopy nicely showed that single VASP tetramers diffuse along the side of actin filaments and confirmed that VASP can track actin filament barbed ends for short times before it dissociates. This last observation explains why efficient processivity requires the formation of clusters of VASP [52]. However, the mechanism by which VASP delivers actin monomers is still unclear since, in vitro, the deletion of the WH2 domain does not abolish the processivity of VASP. In contrast with formins, the importance of profilin for the processive activity of VASP is less clear. Breitsprescher and colleagues found that VASP-mediated processive elongation was not affected by profilin [43], while others found that profilin is required for VASP-mediated processive elongation in high ionic strength conditions [52].

Although the localization of Ena/VASP in FAs suggests a role for these proteins in the formation and dynamics of stress fibers, VASP-deficient cells display normal stress fibers [53]. This observation clearly shows that Ena/VASP proteins do not play a major role in the nucleation of actin filaments that compose stress fibers. Interestingly, the treatment of cells with the proline-rich domain of the *Listeria* protein ActA, that is thought to displace Ena/VASP from FAs [53], affects the incorporation of monomeric actin in FAs [54]. This observation suggests that Ena/VASP control the processive elongation of stress fiber ends anchored to FAs. In addition, the ability of VASP to diffuse along actin filaments could allow the growing actin filament to slip in response to the pulling force exerted by the contractile stress fibers.

### 5.2. Regulation of Actin Assembly by Vinculin

Vinculin is a large actin binding protein of 1066 amino acids present in FAs. Fibroblasts derived from vinculin knockout mice migrate faster, are less adhesive, and exhibit very dynamic FAs [55, 56]. This protein is divided into an N-terminal globular head domain (Vh) and a C-terminal tail domain (Vt) connected by a proline-rich linker (Figure 2). Vinculin exists in a closed inactive conformation in which Vh interacts with Vt and masks the actin filament side binding domain located in Vt. In vitro studies concluded that the binding of one of the numerous vinculin binding sites (VBSs) located throughout talin induces a conformational change of vinculin head that disrupts the Vh-Vt interaction and allows Vt to bind to actin filaments [57]. Whether this mechanism is sufficient to activate vinculin in FAs is still debated [3].

The study of *Shigella* invasion first suggested that vinculin regulates actin dynamics. In this process, the bacterial protein IpaA, that is injected into the host cell, forms a complex with vinculin that blocks the elongation of actin filament barbed ends [58, 59]. More recently, we showed that the Vt domain of vinculin inhibits actin dynamics by blocking barbed end elongation. The domain responsible for the capping activity of Vt is also masked by an autoinhibitory interaction with Vh. Our data also show that actin filament side binding and capping are regulated by distinct mechanisms since the binding of a talin peptide only reveals the side binding but not the capping activity [41] (Figure 3(b)). Vinculin also nucleates actin filaments. However, a significant nucleation activity requires high concentrations of vinculin and nonphysiological ionic strength conditions [41, 60]. It is possible that additional binding partners make vinculin a very potent nucleator in vivo. However, fibroblasts derived from vinculin knockout mice form stress fibers. It is more likely that vinculin-capping activity regulates the elongation of stress fibers. Alternatively, the weak barbed end capping activity of vinculin may allow the slippage of the growing barbed end in a processive-like fashion.

### 5.3. Regulation of Actin Assembly by Formins

Formins are a family of conserved proteins involved in the regulation of microtubule and actin dynamics. In mammals, the formin family is divided into 7 subfamilies: Dia, FHOD, FRL, FMN, INF, DAAM and delphilin. Among them, at least, Dia, FHOD FRL have been shown to play a role in the regulation of actin dynamics associated with cell migration. In particular, the Dia sub-family has been extensively studied. Dia1, 2, and 3 share a characteristic domain organization including a FH1 domain (Formin 1 Homology domain) and a FH2 domain (Formin 2 Homology domain). The FH1 domain contains several proline-rich motifs that interact with profilin. The FH2 domain interacts with actin and nucleates actin filaments [61, 62] (Figure 2). FH1 and FH2 domains tightly cooperate to enhance actin assembly at the barbed end of actin filaments. The FH2 dimer binds to the barbed face of two actin monomers and favors the nucleation of actin filaments. Remarkably, the elongation rate of formin-bound barbed ends from profilin-actin is dramatically enhanced. In this mechanism, the FH1 domain brings profilin-actin complexes to the FH2 domain that is processively “riding” the growing barbed end [63] (Figure 3(c)). Initial studies showed that ATP hydrolysis at the barbed end of the growing actin filament is required for the processive activity of mDia1. In contrast, more recent work described the processive elongation of ADP-actin filaments by mDia1 [62].

Several lines of evidence suggest a role for the formin mDia1 in the regulation of actin assembly associated with FAs. First, the expression of both mDia1 and ROCK induces the formation of stress fibers and FAs [64]. Second, mDia1 is required for the growth of FAs in response to an external force [16]. Third, the knockdown of mDia1 slows down the elongation of dorsal stress fibers in URO cells [34]. Although it is tempting to propose that mDia1 maintains the processive growth of stress fibers in FAs, mDia1 has never been localized to FAs. The formin mDia2 is also involved in actin dynamics associated with FAs. In cells, the injection of inhibitory antibodies directed against mDia2 reduces lamellipodial actin dynamics as well as the number of free barbed ends in FAs. This treatment also reduces focal adhesion disassembly and cell velocity [65]. However, these effects are not direct since mDia2 does not colocalize with the FA signature protein paxillin [65].

The indirect effects of formins on the nucleation and elongation of actin filaments at FAs may reflect the mechanosensitive behavior of FAs. By feeding the lamella with myosin-associated actin filaments (see Section 4.2), formins indirectly increase the contractility of the actin network that is connected to FAs. Hence, the force applied to FAs affects FA turnover and actin dynamics.

**Perspectives**

Recent studies have revealed the mechanisms by which actin-binding proteins regulate actin assembly, the complexity of the myosin II-responsive machinery, and the mechanisms by which a network of contractile cables form to exert a force on the ECM. However, several questions remain open.

The mechanism by which stress fibers processively elongate from FAs is not yet understood. Although the formins mDia1 and mDia2 were considered as good candidates for some time, the effect of their depletion on the elongation of stress fibers is probably indirect. However, it remains possible that other members of the formin family localize in FAs and support the processive elongation of stress fibers. Ena/VASP proteins are also among the candidates. More cell biology observations are needed to attribute such a role to Ena/VASP proteins. This includes FRAP experiments to measure the elongation of stress fibers in cells depleted for the three isoforms of Ena/VASP proteins.

The myosin-II-responsive proteome provides valuable information regarding the machinery involved in the maturation of FAs. However, the detailed description of the sequence of unfolding events occurring in FAs requires the development of new mechanosensors.

Stress fibers are made of a periodic pattern of *α*-actinin and myosin clusters. The force generated by such contractile cables likely depends on the respective size of these clusters. However, the mechanisms by which such a periodic pattern is formed, maintained, and regulated are largely ignored. Does the formation of a periodic pattern result from an autoorganized process or does it involve a machinery including additional F-actin binding proteins and signaling proteins? New concepts for protein segregation may emerge from such a study.
